# Microtubule-associated protein, *MAP1B*, encodes functionally distinct polypeptides

**DOI:** 10.1016/j.jbc.2024.107792

**Published:** 2024-09-19

**Authors:** Tracy C. Tan, Yusheng Shen, Lily B. Stine, Barbara Mitchell, Kyoko Okada, Richard J. McKenney, Kassandra M. Ori-McKenney

**Affiliations:** Department of Molecular and Cellular Biology, University of California, Davis, California, USA

**Keywords:** microtubules, actin, microtubule-associated protein (MAP), kinesin, dynein, DYRK1a, phosphorylation

## Abstract

Microtubule-associated protein, MAP1B, is crucial for neuronal morphogenesis and disruptions in MAP1B function are correlated with neurodevelopmental disorders. *MAP1B* encodes a single polypeptide that is processed into discrete proteins, a heavy chain (HC) and a light chain (LC); however, it is unclear if these two chains operate individually or as a complex within the cell. *In vivo* studies have characterized the contribution of MAP1B HC and LC to microtubule and actin-based processes, but their molecular mechanisms of action are unknown. Using *in vitro* reconstitution with purified proteins, we dissect the biophysical properties of the HC and LC and uncover distinct binding behaviors and functional roles for these MAPs. Our biochemical assays indicate that MAP1B HC and LC do not form a constitutive complex, supporting the hypothesis that these proteins operate independently within cells. Both HC and LC inhibit the microtubule motors, kinesin-3, kinesin-4, and dynein, and differentially affect the severing activity of spastin. Notably, MAP1B LC binds to actin filaments *in vitro* and can simultaneously bind and cross-link actin filaments and microtubules, a function not observed for MAP1B HC. Phosphorylation of MAP1B HC by dual-specificity, tyrosine phosphorylation-regulated kinase 1a negatively regulates its actin-binding activity without significantly affecting its microtubule-binding capacity, suggesting a dynamic contribution of MAP1B HC in cytoskeletal organization. Overall, our study provides new insights into the distinct functional properties of MAP1B HC and LC, underscoring their roles in coordinating cytoskeletal networks during neuronal development.

The morphology of a cell is determined by its underlying cytoskeletal architecture. Disruptions to the establishment or maintenance of cellular morphology, especially neuronal morphology, lead to a range of developmental and degenerative conditions (1). The microtubule and actin cytoskeletons are dynamic polymer networks that act synergistically to regulate cellular shape and intracellular organization. There are numerous microtubule-associated proteins (MAPs) and actin-binding proteins (ABPs) that enable specific polymer architectures and direct either microtubule- or actin-based cargo transport. Growing evidence suggests functionally direct crosstalk between actin and microtubules may be mediated through specialized MAPs and ABPs, but precise mechanisms are disparate and remain unclear (2, 3).

MAP1B is a high molecular weight MAP that was originally identified as one of the most abundant proteins in mammalian brain tissue (4, 5). Both MAP1B and its paralog, MAP1A, are initially translated as single polypeptides. These polypeptides are subsequently cleaved into two distinct components: a heavy chain (HC) and a light chain (LC1 from MAP1B and LC2 from MAP1A) (6, 7, 8). Early studies proposed that the MAP1B and MAP1A HCs could interact interchangeably with LC1, LC2, and also LC3, a protein encoded by a separate gene that is now known to play essential roles in the autophagy pathway (9, 10, 11, 12). However, the observation that MAP1B LC1 exists in 8-fold excess over its corresponding HC in the brain (13) suggests that LC1 and HC might be independently regulated, hinting at possible separate functions within neurons. Later *in vivo* studies further supported this idea, defining independent roles for MAP1B LC1 in actin remodeling during neurite outgrowth, synapse formation, and synaptic transmission (14, 15, 16, 17, 18). Together, these observations raise the question of whether MAP1B HC and LC1 form a functional complex, or if these proteins act separately for distinct cellular purposes.

It is clear from studies using KO mouse models and *in vivo* and *ex vivo* neuronal assays that MAP1B is important for neuronal development from neurite extension to axon branching and dendrite spine maturation to synaptic plasticity and neuronal connectivity (3, 19, 20, 21, 22, 23, 24, 25, 26, 27, 28, 29). It is therefore unsurprising that mutations that disrupt MAP1B production or function result in a range of human neurological conditions (30, 31, 32). However, the molecular mechanisms by which MAP1B contributes to these essential neuronal processes are unknown and the biochemical functions of the HC and LC1 alone or potentially in complex have not been defined.

*In vivo* studies have cataloged several potential molecular actions for MAP1B that involve both the microtubule and actin cytoskeletons. On microtubules, MAP1B has been reported to regulate tubulin posttranslational modifications (25, 33, 34) and microtubule motor transport (18, 35). Strikingly, MAP1B deficiency in mice mimics the phenotype of KIF21A gain-of-function mutants, leading to abnormal development of axons and growth cones in oculomotor nerves and resulting in congenital fibrosis of extraocular muscles type 1 (35). KIF21A is plus-end directed kinesin-4 motor that accumulates at microtubule plus ends to suppress polymerization (36). MAP1B localization on microtubules has also been shown to limit vesicle transport within dendrites, thereby dictating the synaptic insertion of different receptors and overall synaptic transmission (18). Investigations into whether the *in vivo* effects of MAP1B on microtubules motors are direct or indirect are lacking.

Both the MAP1B HC and LC1 contain reported actin-binding domains (ABDs) that are distinct from the microtubule-binding domains (MTBDs) (14, 37, 38). MAP1B has been implicated in regulating actin dynamics during different neuronal processes such as growth cone motility and dendritic spine formation (15, 17, 24). Furthermore, MAP1B has been proposed to mediate microtubule and actin cytoskeletal crosstalk during neuronal polarization (39). To our knowledge, there are no reconstitution studies that have explored the ability of MAP1B HC or LC1 to directly cross-link the microtubule and actin cytoskeletons in a purified system.

Phosphorylation appears to be the predominant regulatory modification of MAP1B HC (40). Two kinases in particular, dual-specificity, tyrosine phosphorylation-regulated kinase 1a (DYRK1A) and glycogen synthase kinase-3 beta, directly phosphorylate MAP1B HC outside of its MTBDs or ABDs during neuronal outgrowth (41, 42, 43). Phosphorylated MAP1B HC is enriched in the distal ends of growing axons, where it appears to regulate actin dynamics and microtubule stability (22, 42, 44, 45). MAP1B HC phosphorylation clearly impacts its cellular activities, but how phosphorylation specifically impacts the biochemistry of MAP1B HC function remains to be determined.

More recent work has highlighted a role for MAP1B in the proliferation or migration of various types of cancer cells including osteoblasts (46, 47), prostate epithelial cells (48), and breast cancer cells (49). The importance of MAP1B in neuronal development, its association with the manifestation of neurological conditions, and its expression in different cancerous cell types underscores the importance of understanding the molecular action of MAP1B in regulating cytoskeletal-based processes.

Despite extensive *in vivo* studies elucidating the phenotypic effects of MAP1B in neurons, the precise molecular mechanisms underlying its interactions with microtubules and actin filaments remain enigmatic. In this study, we present an analysis of the binding behaviors of MAP1B HC and LC1 on microtubules and actin filaments, highlighting distinct characteristics for the HC and LC1 on these two cytoskeletal polymers. We further find that the HC and LC1 do not form a stable biochemical complex, suggestive of independent cellular functions for the two MAP1B chains. MAP1B HC and LC1 similarly inhibit the access of multiple motor proteins to the microtubule lattice, including kinesin-3 (KIF1A), kinesin-4 (KIF21A), and cytoplasmic dynein. In contrast, the HC and LC1 display unique roles in regulating cytoskeletal organization. The MAP1B LC1, but not the HC, is able to promote actin polymerization along microtubules, directly crosslinking these two essential cytoskeletal networks. While the HC binds to actin, it is unable to cross-link microtubules and actin filaments. Additionally, we find that phosphorylation of MAP1B HC by the kinase, DYRK1A, negatively affects its affinity for actin filaments, revealing the importance of phospho-regulation of the MAP1B HC. Our analysis sheds new light on the mechanisms governing the interaction of MAP1B HC and LC1 on microtubules and actin filaments, offering insights into the molecular functions of this critical MAP.

## Results

### MAP1B HC and LC exhibit distinct microtubule-binding behaviors

**Figure 1:**
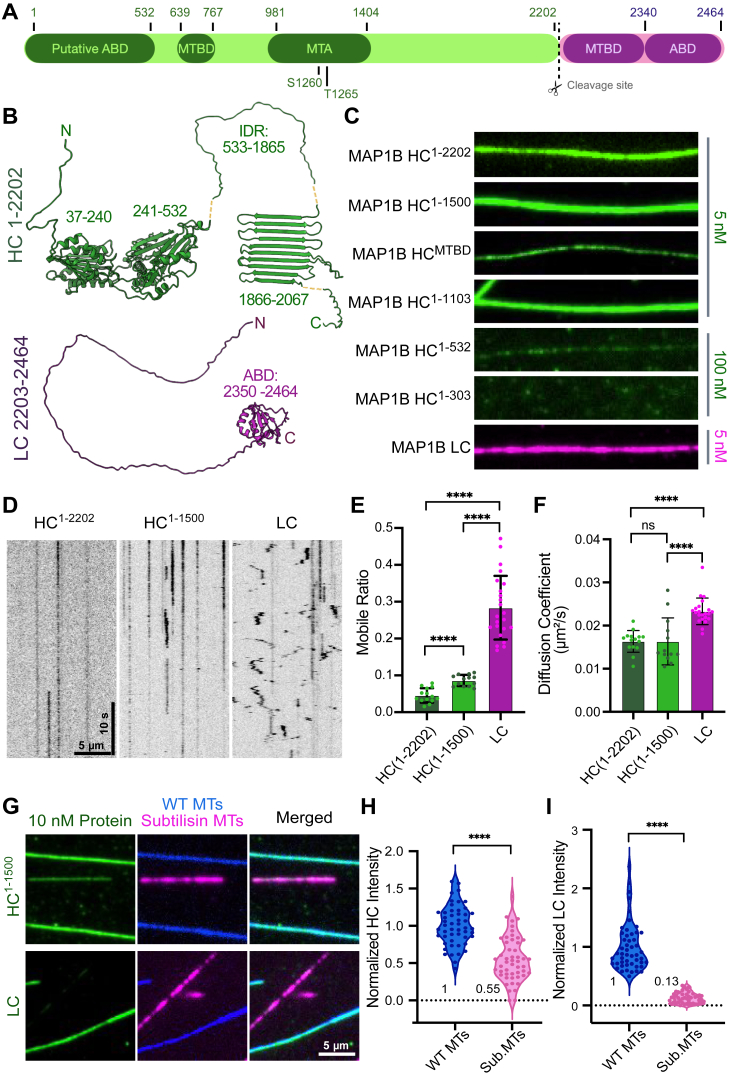
**Analysis of the microtubule-binding behaviors of MAP1B HC and LC.***A,* schematic representation of MAP1B showing the actin-binding domains (ABD) in the HC (aa 1–532) and in the LC (aa 2350–2464), microtubule-binding domains (MTBD) in the HC (aa 639–767) and in the LC (aa 2203–2340), microtubule assembly domain (MTA) in the HC (aa 976–1401), and the reported cleavage site at residue 2202(14). A putative second MTBD in the HC (aa 1–126) has also been reported (53). Two well-characterized phosphorylation sites within the HC are also shown (41). *B,* Alphafold2-Multimer models of MAP1B HC and LC. Residue ranges indicate the start and end of predicted regions. *Yellow dashes* refer to stretches of the protein that are intrinsically disordered but are not shown for simplicity. *C,* TIRF-M images of fluorescently tagged full-length MAP1B HC (aa 1–2202), truncated HC constructs at the indicated amino acid residue range, and MAP1B LC on taxol-stabilized microtubules at indicated concentrations. All images are 15 μm in width. *D,* kymographs of 50 pM MAP1B HC^1-2202^, HC^1-1500^, and LC on taxol-stabilized microtubules from single-molecule assays. *E–F,* quantification of the mobile ratios and diffusion coefficients of 50 pM MAP1B HC^1-2202^, HC^1-1500^, and LC on microtubules. Total analyzed trajectories are n = 622, 645, and 13,914 from n = 13, 15, and 22 movies, respectively, from two independent experiments. ∗∗∗∗ indicates *p* < 0.0001. n.s. indicates *p* = 0.9973. Individual data points are representative of the number of movies. *G,* TIRF-M images of 10 nM MAP1B HC^1-1500^ and LC (*green*) on subtilisin digested (*magenta*) or WT microtubules (*blue*). *H* and *I,* quantification of MAP1B HC^1-1500^ and LC fluorescence intensity on subtilisin-digested microtubules (n = 50) normalized to intensity on WT microtubules (n = 50) from two independent trials. ∗∗∗∗ indicates *p* < 0.0001. All statistical tests were performed with unpaired *t* test. IDR, intrinsically disordered region; C, C terminus; HC, heavy chain; LC, light chain; MAP, microtubule-associated protein; N, N terminus; TIRF, total internal reflection fluorescence.

MAP1B is synthesized as a single polypeptide chain and then proteolytically cleaved into a HC (HC: aa 1–2202, calculated molecular weight: 242.0 kDa) and a LC (LC1: aa 2203–2464, calculated molecular weight: 28.3 kDa; hereafter referred to as LC) (6, 7) (Fig. 1, *A* and *B*). Both the HC and LC reportedly contain nonoverlapping ABDs and MTBDs (14, 38) (Fig. 1*A*). AlphaFold2-Multimer (*via* Colabfold) (50, 51) structural models of the MAP1B HC revealed two globular domains in the N-terminal region spanning residues 27 to 240 and 241 to 532 and an apparent β-meander β-sheet comprised of 10 β-strands (aa 1866–2067). The rest of the HC was predicted to be intrinsically disordered, including the MTBD (aa 639–767) (Fig. 1*B*). The LC models showed an intrinsically disordered N-terminal region, which harbors the MTBD (2203–2349), and a predicted globular domain at the C terminus (2350–2464), which is reportedly the ABD (Fig. 1*B*).

We expressed and purified HC and LC constructs to characterize their microtubule-binding properties using total internal reflection fluorescence (TIRF) microscopy. We first analyzed full-length HC (1–2202, expressed in Sf9 cells), a truncated HC with all the previously defined essential domains (1–1500, expressed in bacteria) and the minimal HC MTBD (aa 639–767, expressed in bacteria) (5, 6, 52). Using a TIRF microscopy-based microtubule-binding assay, we found that HC^1-1500^ exhibited the highest binding affinity among these constructs (K_D_ = 1.8 nM), while the HC^1-2202^ and the MTBD^639-767^ had weaker binding affinities (K_D_ = 3.8 nM and 13.6 nM, respectively) (Figs. 1*C* and S1).

Next, we analyzed three HC constructs corresponding to epilepsy-associated missense variants, which lead to premature stop codons and potential production of truncated HC products: 1 to 303, 1 to 532, and 1 to 1103, all expressed in bacteria (31). HC^1-1103^ had a similar binding affinity to HC^1-1500^(K_D_ = 1.1 nM), while HC^1-303^ and HC^1-532^ exhibited poor binding to the lattice (K_D_ = unstable and 185.0 nM, respectively) (Figs. 1*C* and S1). These results indicate that the secondary microtubule-binding region previously reported within aa 1 to 126 (53) does not robustly interact with microtubules in isolation. Furthermore, the three epilepsy-associated variants have different microtubule-binding affinities, suggesting that the manifestation of the disease may be related to an overall decrease in functional MAP1B protein. The LC (aa 2203–2464) bound with high affinity to microtubules with an apparent K_D_ of 1.5 nM (Figs. 1*C* and S1).

We next examined the single-molecule behaviors of the HC and LC proteins using TIRF microscopy. While the HC^1-2202^ and HC^1-1500^ remained statically bound to the microtubule, the LC diffused on the lattice as evidenced by its significantly increased mobile ratio and diffusion coefficient (Fig. 1, *D*–*F*). To determine the mechanism underlying these differences, we digested microtubules with subtilisin which cleaves the C-terminal tails (CTTs) of microtubules and compared the relative intensity of MAP1B HC and LC proteins on WT *versus* subtilisin-digested microtubules in the same chamber. We observed a 45% decrease in HC^1-1500^ fluorescence intensity on subtilisin-digested microtubules, indicating that the CTTs are not strictly required for the HC–microtubule interaction (Fig. 1, *G* and *H*). Removal of the CTTs largely abolished LC binding as indicated by an 87% decrease in LC fluorescence intensity on subtilisin-digested microtubules (Fig. 1, *G* and *I*). Our data indicate that LC association with the microtubule is dictated by the CTTs to a greater extent than HC association, providing one explanation for the differences in single-molecule behavior we observe between these two MAPs.

### MAP1B HC and LC do not form a robust biochemical complex *in vitro*

**Figure 2:**
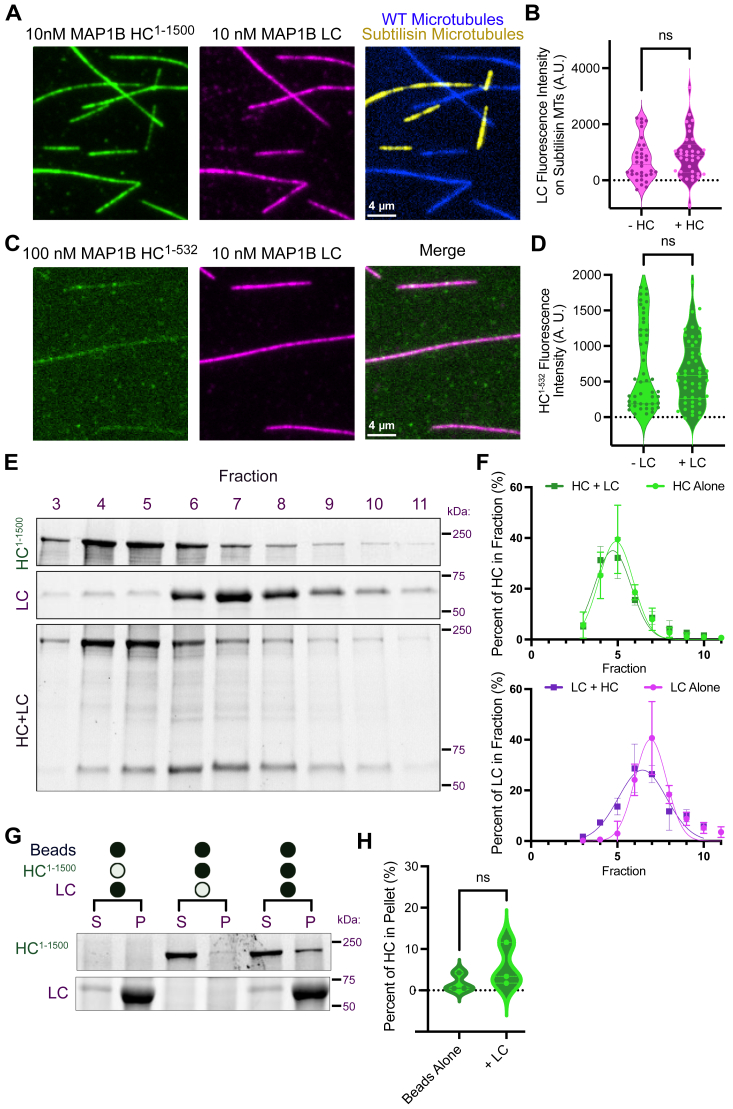
**Recombinant MAP1B HC and LC proteins do not interact biochemically.***A,* TIRF-M images of sfGFP-HC^1-1500^ and LC-mScarlet on WT (*blue*) and subtilisin-digested (*yellow*) microtubules in the same chamber. Scale bars represent 4 μm. *B,* quantification of LC-mScarlet fluorescence intensity on subtilisin-digested microtubules in the absence and presence of HC^1-1500^. n = 34 microtubules per condition from at least n = 2 independent experiments. *p* = 0.2192. *C,* TIRF-M images of sfGFP-HC^1-532^ and LC-mScarlet on microtubules. Scale bars represent 4 μm. *D,* quantification of sfGFP-HC^1-532^ fluorescence intensity on microtubules in the absence and presence of LC. n = 47 (−LC) and 48 (+LC) microtubules from at least n = 2 independent experiments. *p* = 0.4745. *E,* Coomassie Blue–stained SDS-PAGE gels of sfGFP-HC^1-1500^ and LC-mScarlet incubated either alone or together prior to fractionation by size-exclusion chromatography. *F,* quantification of the percent of HC^1-1500^ in each fraction in the absence *versus* the presence of LC, as well as the percent of LC in each fraction in the absence *versus* the presence of HC. n = 3 independent experiments. *G*: Coomassie Blue–stained SDS-PAGE gels of GFP-binding protein (GBP) pull-down assays with purified proteins. LC-sfGFP was pulled down by GBP-conjugated beads. In the absence of LC, mScarlet-HC^1-1500^ is not present in the bead pellet. In the presence of LC, mScarlet-HC^1-1500^ is slightly present in the bead pellet. . Uncropped gels are shown in Figure S4. *H*, quantification of the percent of HC^1-1500^ that pellets with beads in the absence *versus* the presence of LC. n = 3 independent experiments, *p* = 0.3126. All statistical tests were performed with unpaired *t* test. HC, heavy chain; LC, light chain; MAP, microtubule-associated protein; S, supernatant; P, pellet; TIRF, total internal reflection fluorescence.

We next sought to characterize the interaction between the MAP1B HC and LC. Prior work reported that the LC binds within the first 500 amino acids of the HC (6, 11, 13, 14). We investigated this potential interaction both on and off of microtubules. Taking advantage of the fact that the LC cannot bind to subtilisin-digested microtubules while the HC largely retains its ability (Fig. 1*G*), we assayed if HC^1-1500^ was capable of recruiting the LC to subtilisin-digested microtubules *via* a potentially direct HC–LC interaction. However, we found no significant difference in LC intensity on subtilisin-digested microtubules either in the absence or presence of HC^1-1500^ (Fig. 2, *A* and *B*), suggesting that a direct interaction between the HC and LC was not sufficient to recruit the LC to subtilisin-digested microtubules. We then performed a reverse experiment and tested if LC was able to enhance the binding of HC^1-532^ to microtubules. HC^1-532^ has a weak microtubule-binding affinity (Fig. S1) but contains the putative LC-interacting domain. Again, we found no significant difference in HC intensity on microtubules in the absence or presence of LC (Fig. 2, *C* and *D*). To test for an interaction off of microtubules, we used size-exclusion chromatography of purified proteins. We observed no shift in the elution volume for HC^1-1500^ in the presence *versus* the absence of LC, and a small shift for LC in the presence *versus* the absence of HC^1-1500^, indicating a potentially weak interaction (Fig. 2, *E* and *F*). Pull-down assays also revealed an insignificant enrichment of HC^1-1500^ with LC-coated beads compared to beads alone (Fig. 2, *G* and *H*). Similar results were obtained with HC^1-1500^ purified from insect cells (Fig. S2). Together, these data indicate that recombinant MAP1B HC and LC do not form a high-affinity cocomplex either on or off of microtubules.

### MAP1B HC and LC are general inhibitors of motor proteins

**Figure 3:**
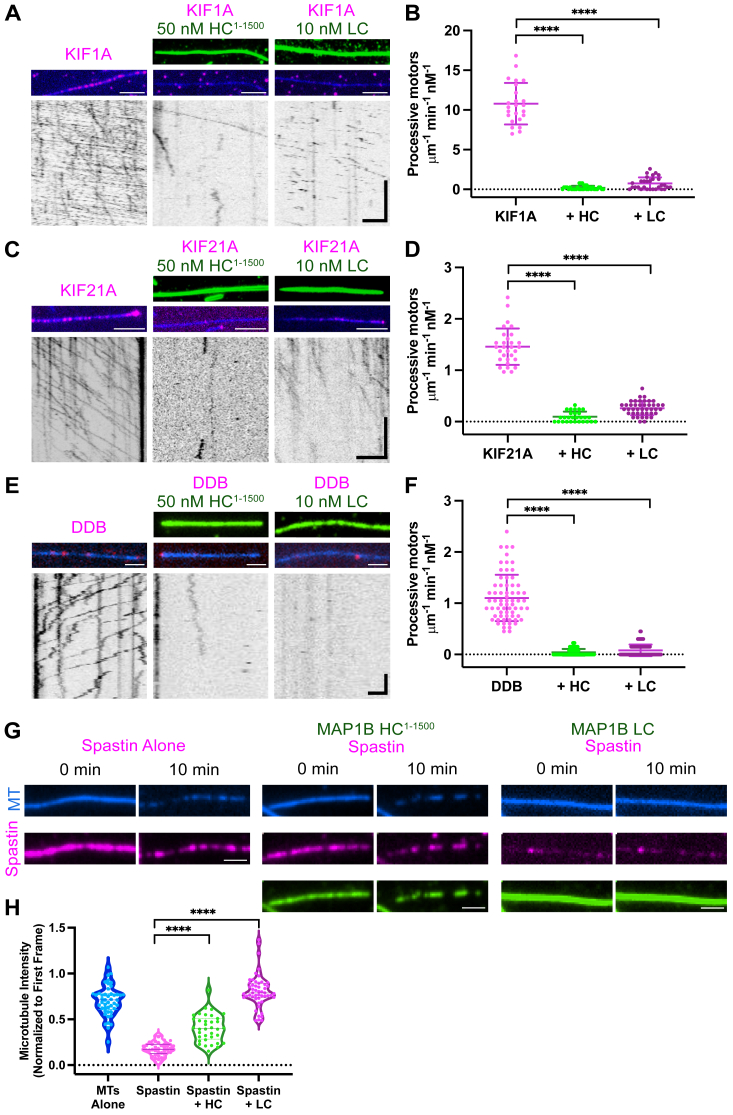
**The effects of MAP1B HC**^**1-1500**^**and LC on motor proteins and spastin.***A,* TIRF-M images and kymographs of 0.25 nM of KIF1A-mScarlet (kinesin-3) + 1 mM ATP in the absence and presence of 50 nM of sfGFP-MAP1B HC^1-1500^ or 10 nM LC-sfGFP. Scale bars: images: 2 μm; kymographs: *x* = 2 μm, *y* = 10 s. *B,* quantification of the number of processive KIF1A motors per μm per minute per nM in the absence and presence of HC^1-1500^ and LC. n = 24, 34, and 30 microtubules, respectively, from n = 2 independent experiments. ∗∗∗∗ indicates *p* < 0.0001. *C,* TIRF-M images and kymographs of 1 nM of KIF21A^1-930^-mScarlet + 1 mM ATP in the absence and presence of 50 nM of sfGFP-MAP1B HC^1-1500^ or 10 nM LC-sfGFP. Scale bars: images: 2 μm; kymographs: *x* = 2 μm, *y* = 10 s. *D,* quantification of the number of processive KIF21A motors per μm per min per nM in the absence and presence of HC^1-1500^ and LC. n = 30, 28, and 42 microtubules, respectively, from n = 2 independent experiments. ∗∗∗∗ indicates *p* < 0.0001. *E,* TIRF-M images and kymographs of 3 nM dynein–dynactin–BicD2(DDB)-TMR + 1 mM ATP in the absence and presence of 50 nM of sfGFP-MAP1B HC^1-1500^ or 10 nM LC-sfGFP. Scale bars: images: 2 μm; kymographs: *x* = 2 μm, *y* = 10 s. *F,* quantification of the number of processive DDB motors per μm per min per nM in the absence and presence of HC^1-1500^ and LC. n = 67, 80, and 88 microtubules, respectively, from n = 2 independent experiments. ∗∗∗∗ indicates *p* < 0.0001. *G,* TIRF-M images of purified, recombinant TMR-spastin either alone or in the presence of 50 nM sfGFP-HC^1-1500^ or 10 nM LC-sfGFP. Scale bars represent 1.5 μm. *H,* quantification of microtubule intensity differences after 10 min alone or after incubation with spastin (n = 40 microtubules), spastin + HC^1-1500^ (n = 29 microtubules), or spastin + LC (n = 50 microtubules) from n = 2 independent experiments, ∗∗∗∗ indicates *p* < 0.0001. All statistical tests were performed with unpaired *t* test. HC, heavy chain; LC, light chain; MAP, microtubule-associated protein; TIRF, total internal reflection fluorescence.

MAP1B is highly expressed in neurons and is localized to both axons and dendrites (20, 23, 27, 29, 40, 45, 54). MAPs are known to regulate the motility of different types of microtubule motor proteins, and we previously found that certain MAPs enable the activity of specific motors (55). We therefore wanted to examine how MAP1B affects different motors on the lattice. We analyzed KIF1A (kinesin-3) and the processive dynein–dynactin–BicD (DDB) complex because these motors transport cargo within both dendrites and axons (56). We also examined KIF21A, a kinesin-4 motor that has previously been reported to interact with MAP1B (35). We found that the numbers of processive KIF1A, KIF21A, and DDB motors on the microtubule were significantly decreased by at least 80% in the presence of either MAP1B HC^1-1500^ or LC (Fig. 3, *A*–*F*). We used HC^1-1500^ for these assays because it contains all the essential elements of the HC and is more easily expressed in bacterial cells, unlike HC^1-2202^. Thus, whether the HC and LC act in a complex or independently, their presence on the microtubule has generally inhibitory effects on KIF1A, KIF21A, and dynein.

### MAP1B HC and LC differentially protect microtubules from severing by spastin

Given that MAP1B HC can still bind subtilisin-digested microtubules while LC binding is abolished on microtubules lacking CTTs (Fig. 1), we investigated how MAP1B HC and LC affect an enzyme that binds and acts upon tubulin CTTs. Spastin is an ATPase that severs microtubules by binding and pulling on the CTTs to destabilize tubulin heterodimer interactions (57). We, and others, have previously showed that tau protects the underlying microtubule lattice from severing by spastin and katanin (58, 59). We performed time-lapse imaging of spastin accumulation and severing of taxol-stabilized microtubules in the absence and presence of MAP1B HC and LC. The fluorescence intensity of microtubules after incubation with spastin for 10 min was reduced by 82%, indicating efficient severing activity (Fig. 3, *G* and *H*). In the presence of MAP1B HC^1-1500^, microtubule fluorescence intensity was reduced by 60%, suggesting partial protection (Fig. 3, *G* and *H*). In the presence of LC, microtubule fluorescence intensity was reduced by only 19% indicating the LC blocks spastin binding and activity to a greater extent than the HC (Fig. 3, *G* and *H*), consistent with the hypothesis that the LC binds directly to the tubulin CTTs. Together, these data show that MAP1B HC and LC are both inhibitors of the motors, KIF1A, KIF21A, and dynein, but have distinct effects on spastin.

### MAP1B LC binds actin and cross-links actin and microtubules

**Figure 4:**
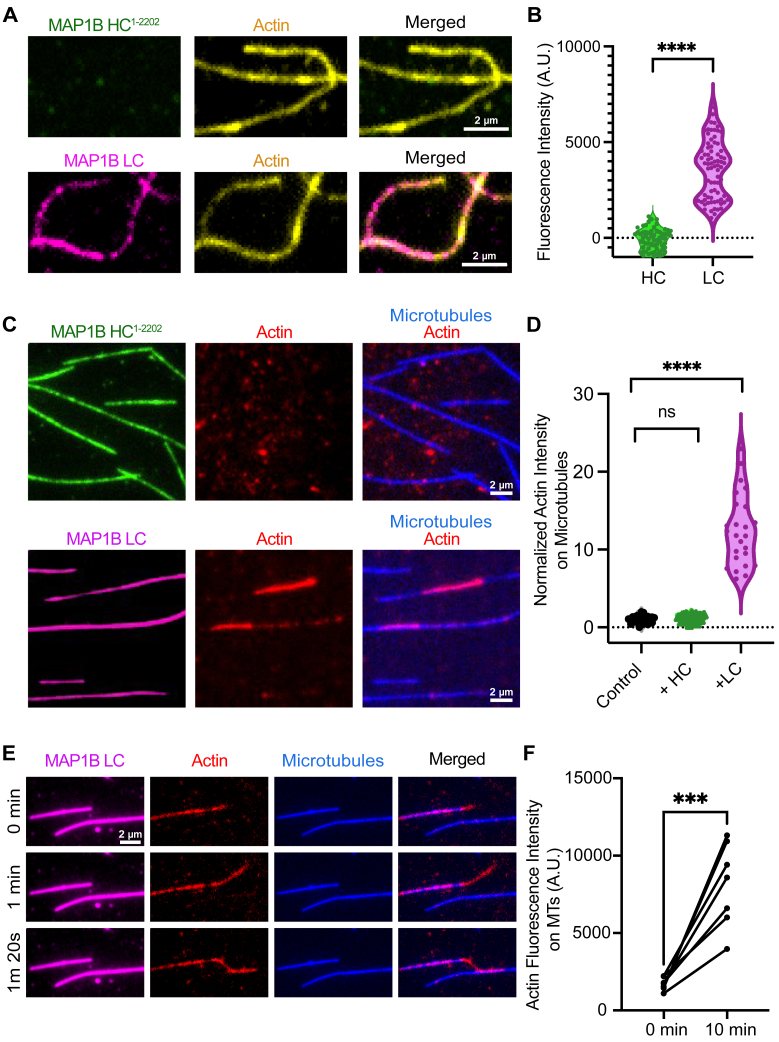
**MAP1B LC associates with actin and cross-links actin filaments and microtubules.***A,* TIRF-M images of 10 nM full-length HC^1-2202^-sfGFP (*green*) and LC-mScarlet (*pink*) on actin filaments (*yellow*). Scale bars represent 2 μm. *B,* quantification of HC^1-2202^ or LC fluorescence intensity on actin filaments. n = 75 and 77 actin filaments for HC^1-2202^ and LC, respectively, from n = 2 independent trials, ∗∗∗∗ indicates *p* < 0.0001. *C,* TIRF-M images of 10 nM MAP1B HC^1-2202^-sfGFP (*green*) or LC-mScarlet (*pink*) on microtubules (*blue*) affixed to the coverslip with actin filaments (*red*) in solution. LC recruits actin filaments to microtubules whereas HC^1-2202^ does not. Scale bars represent 2 μm. *D,* quantification of actin fluorescence intensity on microtubules in the absence or presence of HC^1-2202^ or LC. n = 25 microtubules per condition from n = 2 independent trials, ∗∗∗∗ indicates *p* < 0.0001. n.s. indicates *p* = 0.0618. *E,* TIRF-M images from time-lapse movies of actin growth along microtubules affixed to the coverslip in the presence of 10 nM LC. Scale bars represent 2 μm. *F,* quantification of actin fluorescence intensity on LC-decorated microtubules at 0 min and 10 min. Each line plot represents the average intensity of actin on microtubules in a field of view at 0 min compared to the average intensity of actin on microtubules at 10 min. n = 7 averaged images per time point from n = 2 independent trials. ∗∗∗ indicates *p* = 0.0007. All statistical tests were performed with unpaired *t* test. HC, heavy chain; LC, light chain; MAP, microtubule-associated protein; TIRF, total internal reflection fluorescence.

We observed largely inhibitory effects of MAP1B HC and LC on the enzymatic MAPs we tested, consistent with our previous findings for tau and MAP2 (55, 60, 61). These observations raise the question of why neurons have a diverse set of temporally and spatially overlapping MAPs that inhibit motor activity. What MAP activities would require the absence of cargo transporters and severing enzymes on the microtubule lattice? To explore other potential functions for MAP1B HC and LC, we turned our investigation to their ABDs. Previous studies have detailed roles for both the HC and LC in binding actin (14, 15, 17, 49). We first tested whether MAP1B HC or LC could bind actin filaments *in vitro*. For these assays, we used full length HC^1-2202^ expressed in insect cells, because the ABD has not been as extensively mapped as the MTBD and therefore, we did not want to exclude any other potential ABD regions within the HC. Using TIRF-M, we found that at a concentration of 10 nM, the LC, but not the HC, associated with actin filaments (Fig. 4, *A* and *B*). We transiently transfected BEAS2B cells with MAP1B HC^1-2202^ and LC and imaged their localization patterns upon methanol fixation to preserve the microtubule cytoskeleton or paraformaldehyde fixation to preserve actin stress fibers. Using confocal microscopy, we observed that both MAP1B HC and LC colocalized with microtubules (Fig. S3*A*), but only LC colocalized, albeit weakly, with actin stress fibers (Fig. S3*B*).

Considering the ABD and MTBD of MAP1B LC are located in distinct regions of the molecule, we next asked whether the LC could simultaneously bind both cytoskeletal filaments. We found that MAP1B LC recruited and zippered actin filaments to microtubules affixed to the coverslip, while the HC decorated microtubules, but was unable to recruit actin filaments to the lattice (Fig. 4, *C* and *D*). Using time-lapse imaging in the presence of globular actin (G-actin), we observed actin growth along microtubules in the presence of LC, and when the actin filament extended beyond the end of the microtubule, it was captured by LC bound along an adjacent microtubule (Fig. 4*E*). Interestingly, we did not observe substantial LC accumulation on the actin filaments, indicating the affinity of LC for actin is much lower than the affinity of LC for microtubules. Overall, these results show that MAP1B LC is capable of simultaneously binding microtubules and actin filaments and can therefore cross-link these two cytoskeletal networks and influence the directed growth of actin along microtubules.

### DYRK1a phosphorylation of MAP1B HC negatively regulates its actin-binding activities

**Figure 5:**
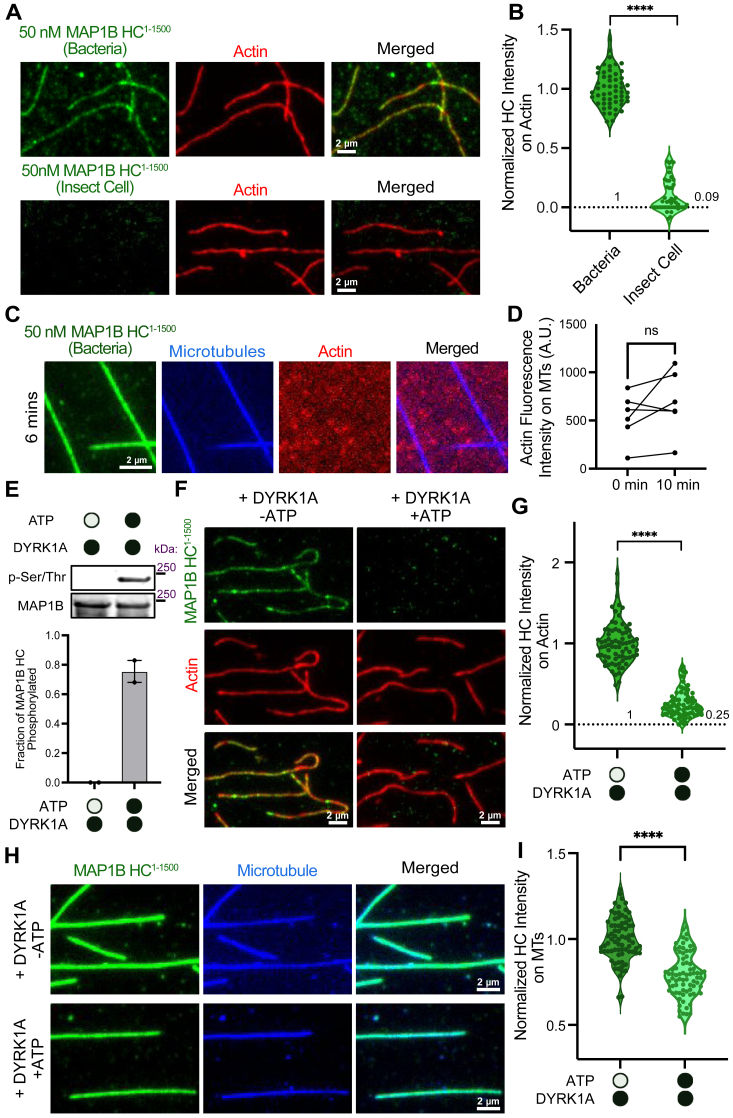
**Phosphorylation of MAP1B HC by DYRK1A regulates its association with actin.***A,* TIRF-M images of 50 nM bacterially expressed sfGFP-MAP1B HC^1-1500^ (not phosphorylated) or Sf9-expressed sfGFP-MAP1B HC^1-1500^ (phosphorylated) (*green*) on actin filaments (*red*). Scale bars represent 2 μm. *B,* quantification of normalized fluorescence intensity of bacterially expressed HC^1-1500^ and Sf9-expressed MAP1B HC^1-1500^ on actin filaments. n = 55 and 48 actin filaments for HC^1-1500^ purified from bacteria *versus* insect cells, respectively, from n = 2 independent experiments, ∗∗∗∗ indicates *p* < 0.0001. *C,* TIRF-M image from a time-lapse movie of actin growth along microtubules affixed to the coverslip in the presence of 50 nM bacterially expressed HC^1-1500^. The scale bar represents 2 μm. *D,* quantification of actin fluorescence intensity on HC^1-1500^-decorated microtubules at 0 min and 10 min. Each line plot represents the average intensity of actin on microtubules in a field of view at 0 min compared to the average intensity of actin on microtubules at 10 min. n = 6 averaged images per time point from n = 2 independent trials. *p* = 0.3875. *E,* Western blots and quantification of the fraction of bacterially expressed strepII-tagged MAP1B HC^1-1500^ phosphorylated by DYRK1a in the absence or presence of 1 mM ATP. The reaction mixtures were subjected to SDS-PAGE, followed by Western blot analysis with α-strepII-tag and α-phospho-serine/threonine antibodies, n = 3 independent experiments and Western blots are plotted on the *bar graph* as individual data points. Uncropped blots are shown in Figure S4. *F*, TIRF-M images of 50 nM of bacterially expressed sfGFP-MAP1B HC^1-1500^ (*green*) after incubation with DYRK1a in the absence or presence of 1 mM ATP on actin filaments (*red*). Scale bars represent 2 μm. *G,* quantification of fluorescence intensity of HC^1-1500^ on actin filaments after incubation with DYRK1a in the absence and presence of 1 mM ATP. n = 76 and 78 actin filaments, for −ATP *versus* + ATP, respectively, from n = 3 independent experiments, ∗∗∗∗ indicates *p* < 0.0001. *H,* TIRF-M images of 50 nM of bacterially expressed MAP1B HC^1-1500^ (*green*) after incubation with DYRK1a in the absence or presence of 1 mM ATP on microtubules (*blue*). Scale bars represent 2 μm. *I,* quantification of fluorescence intensity of MAP1B HC^1-1500^ on microtubules after incubation with DYRK1a in the absence or presence of 1 mM ATP on microtubules. n = 75 microtubules for each condition from n = 3 independent experiments, ∗∗∗∗indicates *p* < 0.0001. All statistical tests were performed with unpaired *t* test. HC, heavy chain; MAP, microtubule-associated protein; TIRF, total internal reflection fluorescence; DYRK1A, dual-specificity, tyrosine phosphorylation-regulated kinase 1a.

We were puzzled that we did not observe actin binding for MAP1B HC^1-2202^ even though it contains both an ABD and a MTBD. We expressed HC^1-2202^ in insect cells due to its insolubility when expressed in bacteria; therefore the purified protein may be phosphorylated by endogenous kinases. Prior studies have suggested that phosphorylation plays a role in regulating HC association with cytoskeletal filaments (38). To investigate if phosphorylation of MAP1B HC affects its association with actin filament, we compared the fluorescence intensity of HC^1-1500^ expressed in bacteria and HC^1-1500^ expressed in insect cells on actin filaments (Fig. 5*A*). Strikingly, bacterially expressed HC^1-1500^ bound to actin filaments while insect cell-expressed HC^1-1500^ did not (Fig. 5, *A* and *B*). However, the bacterially expressed HC^1-1500^ did not cross-link actin filaments to microtubules as we observed for the LC (Fig. 5, *C* and *D*). This indicates that phosphorylation of HC may regulate its association with actin and that simultaneous binding to actin and microtubules may not be possible for the HC.

A prior study showed that the Down syndrome implicated kinase, DYRK1a, specifically and robustly phosphorylates MAP1B HC (41). We therefore performed *in vitro* kinase assays with purified proteins and confirmed that DYRK1a phosphorylates bacterially expressed HC^1-1500^ (Fig. 5*E*). To directly test how DYRK1a phosphorylation of MAP1B affects its actin-binding activities, we analyzed the fluorescence intensity of HC^1-1500^ on actin filaments after incubation with DYRK1a in either the absence or presence of ATP. In the absence of ATP, and therefore the absence of phosphorylation, HC^1-1500^ bound robustly to actin filaments; however, in the presence of ATP, the binding of HC^1-1500^ was reduced by 75% (Fig. 5, *F* and *G*). We next investigated whether phosphorylation of MAP1B by DYRK1a affected its binding to microtubules or if this phospho-switch was specific to actin. Under the same conditions, we found that the fluorescence intensity of HC^1-1500^ was reduced by only 21% upon incubation with DYRK1a and ATP compared to HC^1-1500^ incubated with DYRK1a in the absence of ATP (Fig. 5, *H* and *I*). These results indicate that phosphorylation of MAP1B HC by DYRK1a strongly negates the association of HC with actin, but only mildly alters its ability to bind microtubules.

## Discussion

In this study, we dissected the biophysical properties of the HC and LC proteins encoded by *MAP1B*. We demonstrated that the HC and LC exhibit distinct binding behaviors on microtubules, with LC requiring the tubulin CTTs for efficient binding. Interestingly, we did not observe a significant interaction between HC and LC using various biochemical methods, supporting prior work suggesting these proteins may function independently within cells (13, 14, 16, 17, 18). At saturating concentrations, both HC and LC inhibited three microtubule motors: kinesin-3, kinesin-4, and dynein. However, they differentially affected spastin activity, with LC preventing spastin microtubule severing to a greater extent than HC. Our results also showed that MAP1B LC binds to actin filaments *in vitro* and can simultaneously bind and statically cross-link actin filaments and microtubules. Phosphorylated MAP1B HC was incapable of binding actin filaments, while nonphosphorylated HC-bound actin filaments but could not cross-link microtubules and actin filaments like LC. Additionally, we discovered that DYRK1a phosphorylation negatively regulates the actin-binding activity of MAP1B HC, while having minimal effect on its microtubule-binding capacity. These findings reveal distinct functional properties of the MAP1B HC and LC, providing insight into their roles in organizing cytoskeletal networks within cells.

Our biochemical assays did not detect a robust interaction between MAP1B HC and LC. There is evidence for LC specific roles in regulating actin dynamics and microtubule stability in neurons (13, 16, 17, 18, 62). Combined with prior work, our data indicate that these two chains may not form a constitutive complex. Further experiments are needed to determine if MAP1B HC and LC ever act as a complex in cells, and if so, whether the complex is functional. It is possible that when bound, the HC inhibits LC function or *vice versa*, as previously proposed (13, 14). Although we found that HC purified from either bacteria or insect cells did not significantly interact with LC, there may be modifications other than phosphorylation or adaptors that dictate when and where these two proteins interact. Future work will be necessary to dissect this potential interaction. Based on our results, we propose that HC and LC have separate functions and operate independently within the cell.

Previous studies have shown that MAPs differentially regulate motor proteins and selectively facilitate the motility of different kinesins (55, 60, 61, 63, 64). In contrast, MAP1B impedes various classes of microtubule motors. Both HC and LC broadly inhibited kinesin-3, kinesin-4, and dynein from landing on the microtubule lattice. We therefore classify MAP1B HC and LC with other inhibitory MAPs such as tau and MAP2 (55, 60). Tau has also been shown to cross-link microtubules and actin (65, 66), akin to what we observe here for MAP1B LC. Interestingly, evidence from tau and MAP1A/B KO mouse models suggest MAP1 proteins are functionally redundant with tau (23, 67). Perhaps the similar abilities of tau and MAP1B in inhibiting multiple classes of microtubule motors as well as cross-linking microtubules and actin underlie the observed redundancies.

Given these similarities between tau and MAP1B, we propose that the MAP1B proteins may serve as general inhibitors of motor transport in order to designate the microtubule for another purpose: actin-microtubule cross-linking. Prior work has clearly defined a role for MAP1B in regulating actin-based processes in neurons and proposed that MAP1B mediates crosstalk between actin and microtubules (15, 17, 19, 37). Our current study demonstrates that MAP1B LC directly recruits actin to microtubules, providing the biophysical basis for these prior *in vivo* observations. However, our work identifies LC, not HC, as the key protein performing this static crosslinking function, offering insight into which chain of MAP1B performs this action *in vivo*.

We were surprised to observe that MAP1B HC and LC inhibited KIF21A, given prior work which suggested these two proteins positively interact (35). However, simple genetic analysis supports a model in which MAP1B inhibits rather than promotes KIF21A. In mice, MAP1B loss-of-function mutants mimic the phenotypes found in KIF21A gain-of-function mutants. Ergo, loss of MAP1B equates to activation of KIF21A. This genetic evidence, combined with our biochemical results, supports a model in which MAP1B inhibits KIF21A, and loss of MAP1B function leads to aberrant KIF21A activity.

MAP1B plays a crucial role in neuronal development, and mutations in MAP1B are correlated with the development of a variety of neurological diseases including Parkinson’s, schizophrenia, and periventricular nodular heterotopia (30, 31, 32). Understanding the molecular mechanisms by which MAP1B influences actin and microtubule-based processes is essential for determining the cause and progression of these diseases. We analyzed three epilepsy-associated variants of MAP1B and found that the longest variant (HC^1-1103^) displayed robust microtubule binding, likely due to the presence of the MTBD, while the shorter variants lacking the MTBD, HC^1-532^, and HC^1-303^, did not bind microtubules. The disease phenotypes are similar between these truncations, indicating that the truncated proteins are either unstable and these mutations are hypomorphs or that the common loss of LC in all cases underlies the disease pathology (31). The function of the LC in preventing motor motility and spastin severing, as well as cross-linking the actin and microtubule cytoskeletons may be the key functions that make MAP1B essential for neuronal development.

Our data also reveal that MAP1B HC association with actin is precisely regulated by DYRK1a phosphorylation. Abnormal DYRK1a expression is linked to several neuronal disorders. *DYRK1a* is located on human chromosome 21, and, in trisomy, contributes to the neurological alterations observed in Down syndrome (68, 69, 70, 71, 72, 73). In contrast, recurrent disruptive mutations in *DYRK1a* are correlated with a range of intellectual disability disorders with associated epilepsy (74). Maintaining exact DYRK1a levels is critical for neuronal development, and altering these levels could have pronounced effects on the actin-related activities of MAP1B HC. Given the strong association between MAP1B and neurodevelopmental disorders, altered MAP1B phosphorylation could also be a causative step in the progression of DYRK1a syndromes. Future *in vivo* studies will be necessary to determine the relationship between DYRK1a and MAP1B HC and the downstream consequences of aberrant MAP1B HC phosphorylation.

Overall, we have performed biochemical assays to ascertain the functional roles of MAP1B HC and LC on the microtubule surface and discovered that these two proteins initially linked at translation may indeed separate to perform independent activities within the cell. We hypothesize that MAP1B serves two main physiological roles in neurons. First, we propose that MAP1B LC inhibits microtubule-based motor transport and severing enzyme activity to designate subsets of microtubules for actin-microtubule cross-linking. This idea is supported by *in vivo* evidence demonstrating the necessity of MAP1B LC for actin remodeling during critical neuronal processes (15, 17, 19). Second, we postulate that MAP1B LC and HC restrict microtubule motor activity both during neuronal development and in maintaining cellular homeostasis. We previously identified specific MAPs, MAP7, and MAP9 that facilitate the progression of particular motors on microtubules, arguing that these MAPs need to secure territory on the lattice to ensure efficient motor motility (55, 61). The necessity for specialized functions and spatial organization of MAPs indicates that MAP7 and MAP9 may compete with inhibitory MAPs such as MAP1B, tau, and MAP2. Restricting motor transport is as vital as enabling it, as evidenced by disorders resulting from mutations that disrupt autoinhibition of motor proteins (75). Thus, we propose that a key function of MAP1B LC may be to restrict KIF21A activity, a hypothesis supported by data showing that loss-of-function MAP1B mutations and gain-of-function KIF21A mutations produce similar phenotypes (35). In this way, inhibitory MAPs like MAP1B limit the number of microtubules designated for anterograde and retrograde transport by other MAPs. Our studies into MAP1B function extend our understanding of the MAP code, highlighting the roles of one of the most abundant MAPs in the mammalian brain. Like with any scientific quest, we have traveled along previously defined roads, but also forged new paths that await further expedition. For those interested in exploring MAPs, the continued journey toward discovery is sure to be epic.

## Experimental procedures

### Molecular biology

The complementary deoxyribonucleic acid for protein expression in this study were as follows: mouse MAP1B (Transomic #BC138033), mouse KIF21A (codon-optimized) human spastin (Transomic BC150260), human KIF1A (aa 1–393; Addgene #61665), human DCX (Addgene #83929), and mouse DYRK1a (GE Dharmacon MGC Collection: BC034550). MAP1B HC 1 to 303, HC 1 to 532, HC 1 to 1103, HC 1 to 1500, HC MTBD, and DYRK1a 1 to 499 were cloned in frame using Gibson cloning into a pET28 vector with a N-terminal Strepll-Tag, mScarlet, or a superfolder GFP (sfGFP) cassette. KIF1A (1–393) and MAP1B LC proteins were cloned in frame using Gibson cloning into a pET28 vector with a C-terminal mScarlet-Strepll or sfGFP-Strepll cassette. A fully active, truncated human spastin (Δ227) was cloned into pET28-strepII-SNAPf. MAP1B HC full length and KIF21A 1 to 930 proteins were cloned in frame using Gibson cloning into a pACEBAC1 vector with C-terminal sfGFP-Strepll and mScarlet-Strepll cassette, respectively. HC 1 to 1500 were cloned in frame using Gibson cloning into a pACEBAC1 vector with an N-terminal mScarlet-Strepll cassette.

### Protein expression and purification

For bacterial expression of sfGFP-MAP1B 1 to 303, sfGFP-MAP1B 1 to 532, sfGFP-MAP1B 1 to 1103, sfGFP-MAP1B 1 to 1500, mScarlet-MAP1B 1 to 1500, MAP1B LC 2203-2464-sfGFP, SNAPf-spastin, KIF1A-mScarlet, and KIF21A-mScarlet, BL21-RIPL cells were grown at 37 °C until an absorbance of 0.6 was reached, and protein expression was induced with 0.1 mM IPTG. Cells were grown overnight at 18 °C, harvested, and frozen. BL21 cells expressing DYRK1AΔC (aa 1–499) were grown at 37 °C until an absorbance of 0.6 to 0.8 was reached, and protein expression was induced with 0.2 mM IPTG and allowed to grow for 3 h at 30 °C, and then the cells were harvested and frozen. The cell pellets were rinsed with PBS and resuspended in lysis buffer (50 mM Tris, 150 mM K-acetate, 2 mM MgSO_4_, 2 mM EGTA, 10% glycerol, pH 8.0), supplemented with 1 mM DTT and 1 mM PMSF and protease inhibitor cocktail mix (Roche). Cells were passed through an Emulsiflex press and clarified by centrifugation at 11,000×*g* for 20 min. For insect cell expression of mScarlet-MAP1B 1 to 1500 and MAP1B 1 to 2202-sfGFP, sf9 cells were grown in shaker flask to about 2 million cells/ml and infected at 1:100 ratio of virus and allowed protein expression for 60 h. Cells were harvested and rinsed with PBS. The cell pellet was resuspended in lysis buffer (50 mM Tris, 150 mM K-acetate, 2 mM MgSO_4_, 2 mM EGTA, 10% glycerol, pH 8.0), supplemented with 1 mM DTT, 1 mM PMSF, and protease inhibitor cocktail mix, and then lysed in a dounce homogenizer with 1% Triton X-100. The lysate was centrifuged at 11,000×*g* for 20 min. Clarified lysate from either bacterial or baculovirus expression was passed over a Strep-Tactin XT column (IBA Lifesciences) for 1 h. Protein was washed with lysis buffer for three column volumes and then eluted off from the column with 100 mM desthiobiotin in lysis buffer. Eluted proteins were further purified with an ion exchange column (HiTrap Q HP, Cytiva) or size-exclusion column (Superose-6 GE Health) in lysis buffer on a Bio-Rad NGC system. Peak fractions were collected, concentrated, and flash-frozen in liquid nitrogen. Protein concentration was determined by measuring the absorbance of the fluorescent protein tag and calculated using the molar extinction coefficient of the tag. Purified BicD2N was used to isolate DDB complexes from rat brain cytosol as previously described (76). DDB complexes and spastin were labeled with 5 μM SNAP-TMR dye during the isolation procedure and were frozen in small aliquots and stored at −80 ^o^C.

### Kinase assays

*In vitro* kinase assays were performed with bacterially expressed mScarlet-MAP1B1-1500 and sfGFP-DYRK1aΔC in assay buffer (90 mM Hepes, 50 mM K-acetate, 2 mM EGTA, pH 7.6), supplemented with k-casein, bovine serum albumin (BSA), 10 μM Taxol, and 1 μM phalloidin. 500 nM of bacterially expressed mScarlet-MAP1B HC 1 to 1500 and sfGFP-DYRK1aΔC were incubated in a final volume of 50 uL with or without 2 mM ATP at 37 °C for 30 min. Samples were either run on an SDS-PAGE gel or used in TIRF assays. For the Western blot, the SDS-PAGE gel was transferred to a polyvinylidene difluoride membrane and blotted with antibodies against strepII (Novus NBP2-41076) to detect MAP1B and pan phospho-serine/threonine (Invitrogen MA5-38234) to detect phosphorylation. Both antibodies are commonly used and validated by the commercial company. Blots were imaged on a LiCor Odyssey CLx. Band intensities were analyzed using the Gels Analysis tool in Fiji. For the TIRF assays, the samples were used at the indicated concentrations using the methodology described below.

### Size-exclusion chromatography

Purified MAP1B HC and LC proteins were diluted to 20 μM and incubated alone or together on ice for 1 h, filtered through 0.22 μm filters, then loaded onto a Superose 6 5/150 column in 5 0 mM Tris, 50 mM K-acetate, 2 mM MgSO4, 2 mM EGTA, pH 7.5. Fractions were collected and run on an SDS-PAGE gel. The percent of total protein in each fraction was determined and plotted to determine the distribution of proteins in isolation or upon incubation together.

### Preparation of microtubules and actin filaments

For microtubules, porcine brain tubulin was isolated using a high-molarity Pipes procedure and then labeled with biotin NHS ester, Dylight-405 NHS ester, or Alexa647 NHS ester, as described previously (https://mitchison.hms.harvard.edu/files/mitchisonlab/files/labeling_tubulin_-and_quantifying_labeling_stoichiometry.pdf). A mixture of brain tubulin, biotin-tubulin, and fluorescent-tubulin purified from porcine brain (∼10:1:1 ratio) was assembled in BRB80 buffer (80 mM Pipes, 1 mM MgCl_2_, 1 mM EGTA, pH 6.8 with KOH) with 1 mM GTP for 15 min at 37 °C, and then polymerized microtubules were stabilized with 20 μM taxol and incubated for an additional 20 min at 25 °C. Microtubules were pelleted over a 25% sucrose cushion in BRB80 buffer to remove unpolymerized tubulin, and the pellets were resuspended in BRB80 with 10 μM taxol. For subtilisin removal of the C-terminal tubulin tails, the assembled microtubules were digested with 200 μg/ml subtilisin for 1 h at 37 °C. The digestion was terminated by addition of PMSF, and the digested microtubules were centrifuged over a 25% sucrose cushion to remove the subtilisin protease before use.

For actin filaments, actin was purified from rabbit skeletal muscle acetone powder (Pel-Freez), following established procedures (77). Actin was extracted in G-buffer (2 mM Tris–HCl, 0.1 mM CaCl2, 0.2 mM DTT, and 0.2 mM ATP, pH 8.0) from the acetone powder and subsequently centrifuged using a Fiberlite F14 6 × 250 LE rotor (Thermo Fisher Scientific) at 10,000 rpm for 30 min at 4 °C. The extracted actin was polymerized by supplementing with 50 mM KCl and 2 mM MgCl_2_. After polymerization, the KCl concentration was adjusted to 0.6 M, followed by centrifugation in a Beckman 45 Ti rotor at 45,000 rpm for 2 h at 4 °C. The pelleted actin was dialyzed against G-buffer over 2 days, subsequently spun in a Beckman MLA50 rotor at 50,000 rpm for 20 min at 4 °C, and the resulting supernatant was snap-frozen and stored at −80 °C. For actin labeling, the protein was dialyzed against G-buffer adjusted to pH 7.5, with Tris and DTT substituted by Hepes and tris(2-carboxyethyl)phosphine, respectively. Polymerization was induced by adding 50 mM KCl and 2 mM MgCl_2_, and subsequently, Thermo Fisher Scientific's EZ-Link NHS-PEG4-biotin or DyLight 650 NHS ester were added and further incubated overnight at 4 °C. Labeled actin was sedimented at 80,000 rpm for 20 min in a Beckman TLA100.4 rotor (Beckman Coulter), followed by dialysis against G-buffer, centrifugation, aliquoting, snap-freezing in liquid nitrogen, and storage at −80 °C.

### TIRF microscopy

Flow chambers containing immobilized microtubules were assembled as described (76). Imaging was performed on a Nikon Eclipse TE200-E microscope equipped with an AndoriXon EM CCD camera, a 100X, 1.49 NA objective, four laser lines (405, 491, 568, and 647 nm), and Micro-Manager software (43, micro-manager.org). All binding assays of MAPs on microtubule experiments were performed in assay buffer (90 mM Hepes pH 7.4, 150 mM K-acetate, 2 mM Mg- acetate, 1 mM EGTA, and 10% glycerol) supplemented with 0.1 mg/ml biotin-BSA, 0.5% Pluronic F-168, 10 μM Taxol, and 0.2 mg/ml κ-casein (Sigma). All binding assays of MAPs on actin filaments were performed in assay buffer (90 mM Hepes pH 7., 150 mM K-acetate, 2 mM Mg-acetate, 1 mM EGTA, and 10% glycerol) supplemented with 0.1 mg/ml biotin-BSA, 0.5% pluronic F-168, 10 μM taxol, 1 μM of phalloidin, and 0.2 mg/ml κ-casein (Sigma). ATP concentrations are indicated in the legends. Motility data was analyzed by kymograph analysis in ImageJ (NIH, imageJ.net) to determine landing rates of processive motors. For fluorescent intensity analysis, a segmented line was drawn on microtubule or actin filaments, and the mean intensity value was recorded. The segmented line was then moved adjacent to the microtubules or actin filaments of interest and a local background value was recorded. The background value was subtracted from the value of interest to give the background corrected intensity. For visualizing actin growth in the presence of microtubules and the various MAP1B proteins, after microtubules were affixed to the coverslip, a mixture of 1 μM G-actin (3:1 unlabeled:647-labeled) in the absence or presence of 10 nM LC or 50 nM HC in supplemented assay buffer described above was flowed into the chamber. Images were acquired every 20 s or 1 min for 10 min, and the fluorescence intensity of actin along microtubules was quantified as described above for 0 min and 10 min timepoints.

### Single-molecule analysis for MAP1B

Single-particle tracking was performed using a homemade tracking program written in Matlab as previously described (78). With this advanced single-particle tracking algorithm, we were able to obtain the single-molecule position **r**(t) at time t for MAP1B HC^1-2202^, HC^1-1500^, and LC on taxol-stabilized microtubules, and their trajectories were constructed from the consecutive images. To study the diffusion dynamics of MAP1B, we first selected the mobile trajectories from the whole set of MAP1B trajectories. This is achieved by computing the radius of gyration R_g_(τ) of each MAP1B trajectory obtained over a time period of τ.

$${R_{g}}^{2}\left(\tau \right)=\frac{1}{N}\sum_{i=1}^{N}\left[{\left(x_{i}-\left\langle x\right\rangle \right)}^{2}+{\left(y_{i}-\left\langle y\right\rangle \right)}^{2}\right]\text{,}$$

where N is the total number of time steps in each trajectory, x_i_ and y_i_ are the projection of the position of each trajectory step on the *x-*axis and *y-*axis, respectively, and ⟨x⟩ and ⟨y⟩ are their mean values. Physically, R_g_ quantifies the size of a MAP1B trajectory generated during the time lapse τ. A cut-off value of (R_g_′)_c_ = 0.3 was used in the experiment, below which the MAP1B trajectories are treated as immobile ones. Here, ${R_{g}}^{\prime }=R_{g}/\left\langle R_{g}\right\rangle$ is the normalized radius of gyration, with ⟨R_g_⟩ being the mean value of R_g_. Mean squared displacements, $\left\langle \Delta r^{2}\left(\tau \right)\right\rangle =\left\langle {\left(r\left(t+\tau \right)-r\left(t\right)\right)}^{2}\right\rangle$ were then computed from the mobile trajectories and fitted to $\left\langle \Delta r^{2}\left(\tau \right)\right\rangle =2D_{eff}\tau$ to obtain the effective diffusion coefficients D_eff_ of MAP1B on microtubules.

### Pull-down assays

Pull-down assays were performed with sfGFP-LC, mScarlet-HC^1-1500^ purified from either bacteria or insect cells, and GFP-binding protein (GBP)-conjugated beads. GBP beads were washed into assay buffer (60 mM Hepes pH 7.4, 150 mM K-acetate, 2 mM Mg-acetate, 1 mM EGTA, and 10% glycerol) supplemented with, 0.5% pluronic F-168, and 0.2 mg/ml κ–casein, 1 mM DTT, and 0.1 mg/ml BSA), and then incubated with 500 nM sfGFP-LC or buffer (beads alone control) for 1 h rotating at 4 °C. GBP beads were then washed in assay buffer five times, then resuspended in assay buffer, and 500 nM mScarlet-HC was added to the beads alone control and the experimental condition. The 350 μl final volume solutions were incubated for 1 h rotating at 4 °C. The supernatants were collected, and then the bead pellets were washed five times in assay buffer and resuspended in one bead bed volume. Gel samples of the supernatants and pellets were analyzed by SDS-PAGE gel electrophoresis. The supernatant samples that were run on SDS-PAGE were 15% of the pellet samples. SDS-PAGE gels were stained with Coomassie Brilliant Blue solution and imaged on a BioRad Gel Doc EZ Imager. Band intensities were analyzed using the Gels Analysis tool in Fiji.

### Cell culture and immunocytochemistry

Human lung epithelial BEAS-2B cells (American Type Culture Collection-validated, CRL-9609) were maintained in Dulbecco’s modified Eagle’s medium (Gibco), supplemented with 10% fetal bovine serum, 50 units/ml of penicillin, and 50 μg/ml of streptomycin. All cell cultures were maintained in a 95% air/5% CO_2_ atmosphere at 37 °C. The cell line was routinely confirmed to test negative for *mycoplasma* contamination. The expression vectors used in this study were pEGFP-MAP1B HC 1 to 2202 and pEGFP-MAP1B LC 2203 to 2464. These constructs of MAP1B were cloned into a pEGFP-C1 vector by using Gibson assembly. All constructs were verified by DNA sequencing (GENEWIZ or Plasmidsaurus). Transfections were performed by using the Lipofectamine 2000 reagent kit (Invitrogen) according to manufacturer’s instructions. Cells were generally transfected for 8 h with 1 μg plasmids when the density reached ∼80% confluency. To stain the actin filaments, cells were fixed with 4% paraformaldehyde and permeabilized with 0.2 M NH_4_Cl/PBS-containing 0.2% Triton X-100 for 10 min each at room temperature, and then blocked with 4% BSA (Sigma-Aldrich) in PBS for 2 h. F-actin was stained with Alexa 647–conjugated phalloidin (1:1000, Invitrogen) for 1 h. To stain the microtubules, cells were fixed in −20 °C methanol for 10 min, and then blocked with 4% BSA in PBS at room temperature for 2 h. Next, cells were sequentially incubated with mouse mAbs against α-tubulin (DM1A, 1:500, Sigma-Aldrich) for 1 h, and Alexa Fluor 647–conjugated goat anti-mouse IgG (1:200, Thermo Fisher Scientific) for 1 h. Both the DM1A antibody and Phalloidin stain are well-characterized reagents that have been extensively validated. Cells were washed with PBS (5 min each for three times) before and after the incubation with secondary antibodies, and then mounted onto a microscope slide with ProLong Gold Antifade Mountant (Invitrogen), and then examined by using spinning disk confocal microscopy. All staining and wash processes were performed in room temperature.

### Confocal microscopy

The spinning disk confocal imaging was performed on an inverted research microscope Eclipse Ti2-E with the Perfect Focus System (Nikon), equipped with a Plan Apo 60x NA 1.40 oil objective, a Crest X-Light V3 spinning disk confocal head (Crest-Optics), a Celesta light engine (Lumencor) as the light source, a Prime 95B 25 MM sCMOS camera (Teledyne Photometrics), and controlled by NIS elements AR software (Nikon, www.microscope.healthcare.nikon.com/products/software/nis-elements/). The fluorescent images for BEAS-2B cells were collected over a stack of vertical z-sections across the entire cell ∼4 μm thickness. The final fluorescent images shown in the figures are based on the z-averaged images by using Fiji software (https://fiji.sc/).

### Statistical analysis

All statistical tests were performed with unpaired *t* test.

## Data availability

All data described in the article are contained within the article or are available upon request from Kassandra Ori-McKenney.

## Supporting information

This article contains supporting information.

## Conflict of interest

The authors declare that they have no conflicts of interest with the contents of this article.

## References

[1] Kaufmann W.E., Moser H.W. (2000). Dendritic anomalies in disorders associated with mental retardation. Cereb. Cortex.

[2] Pimm M.L., Henty-Ridilla J.L. (2021). New twists in actin-microtubule interactions. Mol. Biol. Cell.

[3] Mohan R., John A. (2015). Microtubule-associated proteins as direct crosslinkers of actin filaments and microtubules. IUBMB Life.

[4] Vallee R.B., Davis S.E. (1983). Low molecular weight microtubule associated proteins are light chains of MAP 1. Proc. Natl. Acad. Sci. U. S. A..

[5] Bloom G.S., Luca F.C., Vallee R.B. (1985). Microtubule associated protein 1B: identification of a major component of the neuronal cytoskeleton. Proc. Natl. Acad. Sci. U. S. A..

[6] Hammarback J.A., Obar R.A., Hughes S.M., Vallee R.B. (1991). MAP1B is encoded as a polyprotein that is processed to form a complex N-terminal microtubule-binding domain. Neuron.

[7] Togel M., Eichinger R., Wiche G., Propst F. (1999). A 45 amino acid residue domain necessary and sufficient for proteolytic cleavage of the MAP1B polyprotein precursor. FEBS Lett..

[8] Halpain S., Dehmelt L. (2006). The MAP1 family of microtubule-associated proteins. Genome Biol..

[9] Kuznetsov S.A., Gelfand V.I. (1987). 18 kDa microtubule-associated protein: identification as a new light chain (LC-3) of microtubule-associated protein 1 (MAP-1). FEBS Lett..

[10] Schoenfeld T., McKerracher L.M., Obar R.A., Vallee R.B. (1989). MAP 1A and MAP 1B are structurally related microtubule proteins with distinct developmental patterns in the CNS. J. Neurosci..

[11] Mann S.S., Hammarback J.A. (1994). Molecular characterization of light chain 3. A microtubule binding subunit of MAP1A and MAP1B. J. Biol. Chem..

[12] Tanida I., Ueno T., Kominami E. (2008). LC3 and autophagy. Methods Mol. Biol..

[13] Mei X., Sweatt A.J., Hammarback J.A. (2000). Regulation of microtubule-associated protein 1B (MAP1B) subunit composition. J. Neurosci. Res..

[14] Togel M., Wiche G., Propst F. (1998). Novel features of the light chain of microtubule-associated protein MAP1B: microtubule stabilization, self interaction, actin filament binding, and regulation by the heavy chain. J. Cell Biol..

[15] Bouquet C., Ravaille-Veron M., Propst F., Nothias F. (2007). MAP1B coordinates microtubule and actin filament remodeling in adult mouse Schwann cell tips and DRG neuron growth cones. Mol. Cell Neurosci..

[16] Fuhrmann-Stroissnigg H., Noiges R., Descovich L., Fischer I., Albrecht D.E., Nothias F. (2012). The light chains of microtubule-associated proteins MAP1A and MAP1B interact with alpha1-syntrophin in the central and peripheral nervous system. PLoS One.

[17] Henriquez D.R., Bodaleo F.J., Montenegro-Venegas C., Gonzalez-Billault C. (2012). The light chain 1 subunit of the microtubule-associated protein 1B (MAP1B) is responsible for Tiam1 binding and Rac1 activation in neuronal cells. PLoS One.

[18] Palenzuela R., Gutierrez Y., Draffin J.E., Lario A., Benoist M., Esteban J.A. (2017). MAP1B light chain modulates synaptic transmission via AMPA receptor intracellular trapping. J. Neurosci..

[19] Benoist M., Palenzuela R., Rozas C., Rojas P., Tortosa E., Morales B. (2013). MAP1B-dependent Rac activation is required for AMPA receptor endocytosis during long-term depression. EMBO J..

[20] Black M.M., Slaughter T., Fischer I. (1994). Microtubule-associated protein 1b (MAP1b) is concentrated in the distal region of growing axons. J. Neurosci..

[21] Bodaleo F.J., Montenegro-Venegas C., Henriquez D.R., Court F.A., Gonzalez-Billault C. (2016). Microtubule-associated protein 1B (MAP1B)-deficient neurons show structural presynaptic deficiencies in vitro and altered presynaptic physiology. Sci. Rep..

[22] Del Rio J.A., Gonzalez-Billault C., Urena J.M., Jimenez E.M., Barallobre M.J., Pascual M. (2004). MAP1B is required for Netrin 1 signaling in neuronal migration and axonal guidance. Curr. Biol..

[23] Takei Y., Kondo S., Harada A., Inomata S., Noda T., Hirokawa N. (1997). Delayed development of nervous system in mice homozygous for disrupted microtubule-associated protein 1B (MAP1B) gene. J. Cell Biol..

[24] Tortosa E., Montenegro-Venegas C., Benoist M., Hartel S., Gonzalez-Billault C., Esteban J.A. (2011). Microtubule-associated protein 1B (MAP1B) is required for dendritic spine development and synaptic maturation. J. Biol. Chem..

[25] Ziak J., Dorskind J.M., Trigg B., Sudarsanam S., Jin X.O., Hand R.A. (2024). Microtubule-binding protein MAP1B regulates interstitial axon branching of cortical neurons via the tubulin tyrosination cycle. EMBO J..

[26] Diaz-Nido J., Avila J. (1989). Quantitation of microtubule-associated protein MAP-1B in brain and other tissues. Int. J. Biochem..

[27] Gonzalez-Billault C., Engelke M., Jimenez-Mateos E.M., Wandosell F., Caceres A., Avila J. (2002). Participation of structural microtubule-associated proteins (MAPs) in the development of neuronal polarity. J. Neurosci. Res..

[28] Gonzalez-Billault C., Jimenez-Mateos E.M., Caceres A., Diaz-Nido J., Wandosell F., Avila J. (2004). Microtubule-associated protein 1B function during normal development, regeneration, and pathological conditions in the nervous system. J. Neurobiol..

[29] Gonzalez-Billault C., Owen R., Gordon-Weeks P.R., Avila J. (2002). Microtubule-associated protein 1B is involved in the initial stages of axonogenesis in peripheral nervous system cultured neurons. Brain Res..

[30] Walters G.B., Gustafsson O., Sveinbjornsson G., Eiriksdottir V.K., Agustsdottir A.B., Jonsdottir G.A. (2018). MAP1B mutations cause intellectual disability and extensive white matter deficit. Nat. Commun..

[31] Heinzen E.L., O'Neill A.C., Zhu X., Allen A.S., Bahlo M., Chelly J. (2018). De novo and inherited private variants in MAP1B in periventricular nodular heterotopia. Plos Genet..

[32] Julca D.M., Diaz J., Berger S., Leon E. (2019). MAP1B related syndrome: case presentation and review of literature. Am. J. Med. Genet. A..

[33] Bonnet C., Boucher D., Lazereg S., Pedrotti B., Islam K., Denoulet P. (2001). Differential binding regulation of microtubule-associated proteins MAP1A, MAP1B, and MAP2 by tubulin polyglutamylation. J. Biol. Chem..

[34] Utreras E., Jimenez-Mateos E.M., Contreras-Vallejos E., Tortosa E., Perez M., Rojas S. (2008). Microtubule-associated protein 1B interaction with tubulin tyrosine ligase contributes to the control of microtubule tyrosination. Dev. Neurosci..

[35] Cheng L., Desai J., Miranda C.J., Duncan J.S., Qiu W., Nugent A.A. (2014). Human CFEOM1 mutations attenuate KIF21A autoinhibition and cause oculomotor axon stalling. Neuron.

[36] van der Vaart B., van Riel W.E., Doodhi H., Kevenaar J.T., Katrukha E.A., Gumy L. (2013). CFEOM1-associated kinesin KIF21A is a cortical microtubule growth inhibitor. Dev. Cell.

[37] Cueille N., Blanc C.T., Popa-Nita S., Kasas S., Catsicas S., Dietler G. (2007). Characterization of MAP1B heavy chain interaction with actin. Brain Res. Bull..

[38] Pedrotti B., Islam K. (1996). Dephosphorylated but not phosphorylated microtubule associated protein MAP1B binds to microfilaments. FEBS Lett..

[39] Montenegro-Venegas C., Tortosa E., Rosso S., Peretti D., Bollati F., Bisbal M. (2010). MAP1B regulates axonal development by modulating Rho-GTPase Rac1 activity. Mol. Biol. Cell.

[40] Sato-Yoshitake R., Shiomura Y., Miyasaka H., Hirokawa N. (1989). Microtubule-associated protein 1B: molecular structure, localization, and phosphorylation-dependent expression in developing neurons. Neuron.

[41] Scales T.M., Lin S., Kraus M., Goold R.G., Gordon-Weeks P.R. (2009). Nonprimed and DYRK1A-primed GSK3 beta-phosphorylation sites on MAP1B regulate microtubule dynamics in growing axons. J. Cell Sci..

[42] Trivedi N., Marsh P., Goold R.G., Wood-Kaczmar A., Gordon-Weeks P.R. (2005). Glycogen synthase kinase-3beta phosphorylation of MAP1B at Ser1260 and Thr1265 is spatially restricted to growing axons. J. Cell Sci..

[43] Tymanskyj S.R., Lin S., Gordon-Weeks P.R. (2010). Evolution of the spatial distribution of MAP1B phosphorylation sites in vertebrate neurons. J. Anat..

[44] Owen R., Gordon-Weeks P.R. (2003). Inhibition of glycogen synthase kinase 3beta in sensory neurons in culture alters filopodia dynamics and microtubule distribution in growth cones. Mol. Cell Neurosci..

[45] Villarroel-Campos D., Gonzalez-Billault C. (2014). The MAP1B case: an old MAP that is new again. Dev. Neurobiol..

[46] Peng L., Zhang D., Tu H., Wu D., Xiang S., Yang W. (2024). The role of Map1b in regulating osteoblast polarity, proliferation, differentiation and migration. Bone.

[47] Su P., Tian Y., Yin C., Wang X., Li D., Yang C. (2022). MACF1 promotes osteoblastic cell migration by regulating MAP1B through the GSK3beta/TCF7 pathway. Bone.

[48] Wang Z., Zhang Y., Zhao C., Li Y., Hu X., Wu L. (2021). The miR-223-3p/MAP1B axis aggravates TGF-beta-induced proliferation and migration of BPH-1 cells. Cell Signal.

[49] Inoue H., Kanda T., Hayashi G., Munenaga R., Yoshida M., Hasegawa K. (2024). A MAP1B-cortactin-Tks5 axis regulates TNBC invasion and tumorigenesis. J. Cell Biol..

[50] Mirdita M., Schutze K., Moriwaki Y., Heo L., Ovchinnikov S., Steinegger M. (2022). ColabFold: making protein folding accessible to all. Nat. Methods.

[51] Jumper J., Evans R., Pritzel A., Green T., Figurnov M., Ronneberger O. (2021). Highly accurate protein structure prediction with AlphaFold. Nature.

[52] Pedrotti B., Islam K. (1995). Microtubule associated protein 1B (MAP1B) promotes efficient tubulin polymerisation in vitro. FEBS Lett..

[53] Gomi F., Uchida Y. (2012). MAP1B 1-126 interacts with tubulin isoforms and induces neurite outgrowth and neuronal death of cultured cortical neurons. Brain Res..

[54] Meixner A., Haverkamp S., Wassle H., Fuhrer S., Thalhammer J., Kropf N. (2000). MAP1B is required for axon guidance and Is involved in the development of the central and peripheral nervous system. J. Cell Biol..

[55] Monroy B.Y., Tan T.C., Oclaman J.M., Han J.S., Simo S., Niwa S. (2020). A combinatorial MAP code dictates polarized microtubule transport. Dev. Cell.

[56] Lipka J., Kapitein L.C., Jaworski J., Hoogenraad C.C. (2016). Microtubule-binding protein doublecortin-like kinase 1 (DCLK1) guides kinesin-3-mediated cargo transport to dendrites. EMBO J..

[57] Roll-Mecak A., McNally F.J. (2010). Microtubule-severing enzymes. Curr. Opin. Cell Biol..

[58] Tan R., Lam A.J., Tan T., Han J., Nowakowski D.W., Vershinin M. (2019). Microtubules gate tau condensation to spatially regulate microtubule functions. Nat. Cell Biol..

[59] Siahaan V., Krattenmacher J., Hyman A.A., Diez S., Hernandez-Vega A., Lansky Z. (2019). Kinetically distinct phases of tau on microtubules regulate kinesin motors and severing enzymes. Nat. Cell Biol..

[60] Siahaan V., Tan R., Humhalova T., Libusova L., Lacey S.E., Tan T. (2022). Microtubule lattice spacing governs cohesive envelope formation of tau family proteins. Nat. Chem. Biol..

[61] Monroy B.Y., Sawyer D.L., Ackermann B.E., Borden M.M., Tan T.C., Ori-McKenney K.M. (2018). Competition between microtubule-associated proteins directs motor transport. Nat. Commun..

[62] Lerch-Gaggl A.F., Sun K., Duncan S.A. (2007). Light chain 1 of microtubule-associated protein 1B can negatively regulate the action of Pes1. J. Biol. Chem..

[63] Chaudhary A.R., Lu H., Krementsova E.B., Bookwalter C.S., Trybus K.M., Hendricks A.G. (2019). MAP7 regulates organelle transport by recruiting kinesin-1 to microtubules. J. Biol. Chem..

[64] Balabanian L., Lessard D.V., Swaminathan K., Yaninska P., Sebastien M., Wang S. (2022). Tau differentially regulates the transport of early endosomes and lysosomes. Mol. Biol. Cell.

[65] Elie A., Prezel E., Guerin C., Denarier E., Ramirez-Rios S., Serre L. (2015). Tau co-organizes dynamic microtubule and actin networks. Sci. Rep..

[66] He H.J., Wang X.S., Pan R., Wang D.L., Liu M.N., He R.Q. (2009). The proline-rich domain of tau plays a role in interactions with actin. BMC Cell Biol..

[67] Harada A., Oguchi K., Okabe S., Kuno J., Terada S., Ohshima T. (1994). Altered microtubule organization in small-calibre axons of mice lacking tau protein. Nature.

[68] Altafaj X., Dierssen M., Baamonde C., Marti E., Visa J., Guimera J. (2001). Neurodevelopmental delay, motor abnormalities and cognitive deficits in transgenic mice overexpressing Dyrk1A (minibrain), a murine model of Down's syndrome. Hum. Mol. Genet..

[69] Dowjat K., Adayev T., Kaczmarski W., Wegiel J., Hwang Y.W. (2012). Gene dosage-dependent association of DYRK1A with the cytoskeleton in the brain and lymphocytes of down syndrome patients. J. Neuropathol. Exp. Neurol..

[70] Fenster R., Ziegler A., Kentros C., Geltzeiler A., Green Snyder L., Brooks E. (2022). Characterization of phenotypic range in DYRK1A haploinsufficiency syndrome using standardized behavioral measures. Am. J. Med. Genet. A..

[71] Guedj F., Pereira P.L., Najas S., Barallobre M.J., Chabert C., Souchet B. (2012). DYRK1A: a master regulatory protein controlling brain growth. Neurobiol. Dis..

[72] Ori-McKenney K.M., McKenney R.J., Huang H.H., Li T., Meltzer S., Jan L.Y. (2016). Phosphorylation of beta-tubulin by the down syndrome kinase, minibrain/DYRK1a, regulates microtubule dynamics and dendrite morphogenesis. Neuron.

[73] Wegiel J., Dowjat K., Kaczmarski W., Kuchna I., Nowicki K., Frackowiak J. (2008). The role of overexpressed DYRK1A protein in the early onset of neurofibrillary degeneration in Down syndrome. Acta Neuropathol..

[74] van Bon B.W., Coe B.P., Bernier R., Green C., Gerdts J., Witherspoon K. (2016). Disruptive de novo mutations of DYRK1A lead to a syndromic form of autism and ID. Mol. Psychiatry.

[75] Chiba K., Takahashi H., Chen M., Obinata H., Arai S., Hashimoto K. (2019). Disease-associated mutations hyperactivate KIF1A motility and anterograde axonal transport of synaptic vesicle precursors. Proc. Natl. Acad. Sci. U. S. A..

[76] McKenney R.J., Huynh W., Tanenbaum M.E., Bhabha G., Vale R.D. (2014). Activation of cytoplasmic dynein motility by dynactin-cargo adapter complexes. Science.

[77] Spudich J.A., Watt S. (1971). The regulation of rabbit skeletal muscle contraction. I. Biochemical studies of the interaction of the tropomyosin-troponin complex with actin and the proteolytic fragments of myosin. J. Biol. Chem..

[78] Shen Y., Ori-McKenney K.M. (2024). Microtubule-associated protein MAP7 promotes tubulin posttranslational modifications and cargo transport to enable osmotic adaptation. Dev. Cell.

