# Supplementary material for: Microtubule-associated protein, MAP1B, encodes functionally distinct polypeptides
Source: J Biol Chem. 2024 Sep 19;300(11):107792. doi: 10.1016/j.jbc.2024.107792 (PMC11530598; doi:10.1016/j.jbc.2024.107792)
Supplement: Supporting Information [file mmc1.docx]

**Microtubule-associated protein, *MAP1B*, encodes functionally distinct polypeptides**

**Tan *et al.***

**Supporting Figures and Figure Legends**

**Figure S1. Saturation curves for purified recombinant proteins used in this study. Related to Figure 1.** Quantification of fluorescence intensity of microtubule-bound sfGFP-MAP1B constructs plotted against concentration. The K_D_ for each protein is indicated. For MAP1B HC^1-303^, a K_D_ could not be determined due to poor binding even at high protein concentrations.

**Figure S2. MAP1B HC and LC do not form a robust biochemical complex. Related to Figure 2.** Coomassie Blue-stained SDS-PAGE gel of GFP-binding protein (GBP) pull-down assays with purified proteins. sfGFP-LC was pulled down by GBP-conjugated beads. mScarlet-HC^1-1500^ expressed in insect cells displays a similar level of interaction with LC compared to mScarlet-HC^1-1500^ expressed in bacteria (Figure 2G). S = supernatant and P = pellet.

**Figure S3. Localization patterns of MAP1B HC and LC expressed in BEAS2B cells. Related to Figure 4.** Confocal images of the human epithelial cell line, BEAS2B, expressing sfGFP tagged MAP1B HC^1-2202^ or LC, and immunostained for tubulin using DM1A (A) or actin using Phalloidin (B). Insets show zoomed in regions of the cytoskeletal filaments. Scale bars: 5 μm for large images and 2 μm for insets. n = three independent experiments for all conditions.

**Figure S4. Uncropped images of SDS-PAGE gels shown in Figure 2E and 2G and blots shown in Figure 5E.** Red boxes highlight the portions of the gels or blots that are shown in the figure. Ladder is shown on the left. (A) Uncropped gels for Figure 2E. (B) Uncropped gel for Figure 2G. (C) Uncropped blots for Figure 5E.
